# Notes of a Visit to Lunatic Asylums in Great Britain and Ireland

**Published:** 1860

**Authors:** Joseph Workman


*Notes of a Visit to Lunatic Asylums in Great Britain and Ireland.* By JOSEPH WORKMAN, M. D. From a Report to the Visiting Commissioners of the Provincial Lunatic Asylum, at Toronto, C. W.

I commenced my professional tour by visiting the lunatic asylum of the West Riding, at Wakefield.

This institution was first opened for reception of patients in November, 1818, and from that time to 1st January, 1859, had given admission to 7,045 cases of lunacy, of which 2,986 resulted in recovery, 633 in relief of condition, and 2,456 in death—leav-880 remaining in at the latter date, which number has since, by the opening of a contiguous branch asylum, been increased to 950.

The chief asylum consists of two distinct buildings, both of which are complete as to means of classification; but the new erection is much superior to the old in its internal arrangements.

The grounds contain 66 acres, part of which is laid out in plantings, shrubberies, flower-beds, gardens and orchard, and the remainder as a farm, in a high state of cultivation, exclusively by spade labor. Good order, cheerfulness, industry, comfort and kindness appeared to pervade the entire establishment. The medical superintendent, Dr. Cleaton, is a gentleman of superior qualifications, and he seems to have infused his good spirit into the whole institution. The Committee of Visitors have most liberally responded to his large requisitions for pecuniary aid, and have at present in progress several large and expensive works, for the extension and improvement of the establishment, among which I may mention a very large common dining-hall, for 600 patients of both sexes, with a gallery for seating the same at morning and evening prayers; also, a large central kitchen, contiguous to the dining hall, to be furnished with a complete new cooking apparatus, on the most approved plan; and thirdly, a commodious and beautiful chapel, at a short distance from the asylum, on a suitable site. The contract for this building is over £4000 sterling. The outbuildings comprise gas-works, brewery, bakery, engine-house, farm-houses, shops for various trades, laundry and extensive appendages, and, in short, every other convenience, which, in England, is considered necessary to form a complete public institution on a magnificent scale. On the occasion of my visit, a ball was given to the patients in honor of their Canadian friend; and I had the pleasure of seeing 150 of them enjoy themselves in appropriate dances, with such gratification and propriety as could not fail to interest the most fastidious observer, and certainly made me feel quite at home.—The evening's amusements were closed by the whole company singing the National Anthem, in such a style and with such enthusiasm as only in England could be witnessed.

Everything in this institution excited my admiration, and commanded approval; but everything told me of much to be done at home to bring our institution to the mark of excellence which was here before me. The Wakefield Asylum presents the most ample means of classification of the patients—there are 12 wards for men, and 14 for women. In this advantage, I could readily perceive, lay the explanation of the admirable condition and excellent discipline of the establishment; verifying most decisively the fact, that the chief difficulty of governing a lunatic asylum is not found in the large number of its inmates, but in the absence of their thorough classification.

The diet, clothing, bedding, and all other necessaries and comfarts of this asylum, are on the most liberal scale. The patients have Sunday dress distinct from their week-day working clothes, and beer is a regular beverage. The working parties are large, and the amount of labor done is considerable. Concerts, balls, pic-nic excursions, and various other indulgences, are freely given, and are found very beneficial. Indeed, I may say that the pauper inmates of the Wakefield Asylum are provided for in such a manner as to inspire a stranger visiting it, with the very highest opinion of the people at large; for only among a great and good people could such an institution exist.

I must not omit to mention a highly important circumstance connected with this institution, which I also found to obtain in some others. I allude to the system of free and well-regulated social intercourse between the male and female patients. Dr. Cleaton has had extensive opportunity of observation in this and other large asylums; and he feels convinced that much benefit is derived from this social regulation. It is with this view that the capacious dining-hall, above noticed, which will also serve as a concert and ball-room, has been decided on; and that various other arrangements are made to bring the male and female patients frequently together. As a contrary principle has been advocated in America, and has actually been inaugurated in Pennsylvania, it is to be hoped a full trial of the Wakefield system may arrest the progress of theoretic innovation.

A large extent of stone flooring in the Wakefield old asylum has been taken up, and replaced by wood, as more comfortable and less dangerous and troublesome. The old high boundary walls, of prison character, are in progress of removal, and a low wall, surmounted by an iron railing, enclosing enlarged grounds, is to replace it.

Whilst at Wakefield, I took the opportunity of visiting the prison, which is an extensive and admirable institution, containing over 1000 prisoners, 500 of whom are convicts from various parts of the kingdom. The institution consists of two distinct buildings, an old prison and a new one. The latter is in every way superior to the former. Dr. Milner, the prison-surgeon, gave me much information on those subjects on which I sought it. I have never seen a more clean, or a better ordered institution, than the new prison. It is said to be one of the best in England.

Ventilation is effected by rarefying towers, with fires in them, above the level of the uppermost cells. Into these shafts the impure air is conducted by converging flues coming from the various apartments beneath. As the prisoners are constantly confined at work in their respective cells, artificial ventilation is indispensable; and the state of the cells of the new prison, compared with that of the cells of the old one, in which the ventilating towers are wanting, sufficiently demonstrated the value of the provision. The dimensions of the cells are about 14 feet long, 7 feet wide, and 9 feet high; giving over 900 cubic feet to each prisoner; which meets the Government requirement. The mortality is only 14 persons per 1000 annually.

The next public institution which I visited was the asylum at York, for the North and East Ridings, under charge of Dr. Hill.

The building is two stories high, with basement, and is 600 feet in length, with appropriate wings attached. It contains 440 patients; the grounds contain 160 acres of good land, well cultivated, and the gardens and shrubberies are neat and extensive.—The patients were more noisy, and apeared less comfortable, than at Wakefield. The matron is the wife of the physician, a regulation expedient in some asylums of the old country, from two circumstances: 1st. The giving of too high a salary to the matron; £150 in this institution, and more in some others. 2nd. Placing in the office, for the sake of the living, women of reduced circumstances, and previous high position, who are unqualified for the duties, and incapable of learning them. The result has invariably been formidable antagonism and very defective administration. The remedy for these evils has been found in bestowing the matronship on the medical superintendent's wife, who, if she has no family, and is a woman of humble mind, and great energy, may work harmoniously, but hardly subordinately.

Several large associated dormitories are found in this asylum, with 40 beds in each. The great bulk of the patients are incurables. In this institution, and some others, cattle are purchased and fed for slaughter, for the supply of the house, and thus superior meat is obtained at a low price. The gas used here is supplied by the city works.

I observed one or two cases of sanguineous ear-tumor, in this asylum. I have, for some time, had my own suspicion as to the source of this malady. It has latterly been very unfrequent in the Toronto Asylum. It is rarely, or almost never, met with among females. This circumstance has led me to conjecture that it has some relation to short hair. Some very interesting papers on this affection have been read at the annual meetings of American Superintendents. The writers have regarded it as purely idiopathic and peculiar to the insane. I am unable to concur in this opinion.

The celebrated *Retreat*, at York, an Asylum established and sustained by the amiable sect of Quakers, next claimed attention. It is an admirable institution, and is conducted on the same mild and benevolent system by which it has ever been distinguished. It contains at present 110 patients, many of whom have superior accommodations, and pay from two to three guineas a week.—Those who are unable to pay, are supported from the over-charges of the rich; and the rule is cheerfully complied with. This asylum well merits the high reputation which it holds. The ventilation appeared to me to be defective, owing, as I thought, to the profusion of trees and shrubberies which closely envelope the house, and prevent free ingress of air and light, an evil too common both in England and America. The nurses in this asylum are paid from ten to fourteen guineas a year, and other servants in proportion. The service of the institution is very efficient, and the discipline is exact. The patients are indulged in various amusements and games, but I heard nothing of dancing; yet no other recreation is better suited to the mental and bodily improvement of the insane.

From York, I returned southward through Sheffield to Derby, where I found a new asylum, constructed on the modern plan of English asylum architecture; and under charge of one of the ablest superintendents in the Kingdom, Dr. Hitchman, formerly of the Hanwell Asylum, London. The grounds of the asylum contain 69 acres, presenting, perhaps, the most beautiful site in all England. I know not any higher terms in which to express my conviction of their loveliness. The buildings, furniture, &c., &c., and the land, have cost £98,396 sterling. The institution was intended to contain 300 patients, but has, as yet, only 270. It may, therefore, be regarded as a very expensive one, but it accords with the present requirements of the English Commissioners in Lunacy. I beg to refer your Board to the "Derby Asylum Report" for 1853, for a view of the ground-plan and elevation, which I herewith lay before you.

Pictures, statuary, flowers, singing birds, pet animals, and various other objects of beauty and interest, are abundantly placed in every ward, and the superintendent's apartments are elegantly furnished. Heating is effected by hot water and by fire-grates, and ventilation by rarefying towers; both are expensive, and I fear inefficient. The rarefying towers are heated at the bottom, and not, as in the Wakefield prison, at the top. This is the same error that was committed in the Toronto asylum. The heating by hot water is effected in basement chambers, and not, as in the Toronto Asylum, by radiation in the rooms supplied.—It is therefore defective and irregular. The garden, shrubberies and farms are in the highest cultivation; and the farm-stock is perhaps the best in England. Gas-works, steam-engine, bakery, brewery, and laundry, and every other appurtenance, are of the best construction.

The universal comfort, cleanness, and good order of this asylum, not only commanded my admiration, but astonished me.—I felt that in Canada we have a great deal to do before we can flatter ourselves as having approximated to perfection. The Derby Asylum has six wards for each sex, with outside enclosed, and ornamented airing-courts corresponding. There is no crowding, and the means of classification are ample; and besides, there is no shortness of funds with which to accomplish all that is here seen or desired. I very much fear that, in Canada, any Board of Governors establishing and supporting an institution in the style of the Derby Asylum, would be very severely criticised by that class of public benefactors who make capital from their sympathy with our over-taxed people; and yet this is a pauper asylum.

I left this institution and its accomplished superintendent with mingled feelings of regret and esteem, regarding myself as well paid for my voyage and journey, though I should see nothing else in the old world.

From Dirby I proceeded to Birmingham, and there inspected the Borough Asylum, contiguous to this fine town.

It falls short, in many respects, of that at Derby, though in structure resembling it. The grounds amount to only 20 acres. The number of patients is 364. It is overcrowded; and the Commissioners in Lunacy refuse permission to enlarge it, unless more land is added to the grounds. The corporation are niggardly, and refuse further outlay for land; but the requirement of the Commissioners will be enforced, and most properly; for nothing is of greater value than sufficiency of land for a lunatic asylum. It is to be wished this fact was as well known in Canada as it is in England.

The borough jail, work-house, and this asylum, are all under the same corporate, fiscal control, and visitorial government; and thus is accounted for the inferior condition of the last-named institution. Lunatic asylum government should not be associated with that of prisons and poor-houses.

I next visited the Warwick Asylum. It differs from that of Derby, chiefly in having its wings carried to the front, instead of the rear, and in being less ornamental. An artesian well has been obtained by boring 250 feet. Heating and ventilating, I learned, are defective. In the climate of Canada they would be still more so. The patients had a festive entertainment out of doors, on the day before my visit, and the people of the good old town of Warwick had joined in the sports. These indulgences seem to be well understood in England, where the rich are not too proud to find pleasure in seeing the poor made happy. Some of the patients had danced rather freely, and were languid from the fatigue, and perhaps from indulgences of a more national and substantial character. Dr. Parsey, the superintendent, was very courteous and attentive.

Having thoroughly inspected this asylum, I proceeded to London, where I first visited the Asylum of Bethlem Hospital, a very handsome building, with limited but beautiful grounds.— This institution contains at present a considerable number of respectable inmates of reduced circumstances, and unfortunately a large number, besides, of a different class, that is to say, criminal lunatics. It is impossible to carry into effect, in such an institution, that benign system of administration which is practicable in asylums of a different order of population. Here are to be found some of the worst characters which the immence city of London can furnish; men whose criminal life has led to insanity; but mixed with these, many whose insanity has prompted to crime; and, occasionally, are presented a few who are worse than either—impostors, who, to screen themselves from just punishment, have simulated insanity. A national criminal asylum will soon be opened to receive the criminal insane of England.— Its completion will be a happy era in the history of insanity. It will, no doubt, be conducted on benevolent but judicious principles. The total number of patients in Bethlem is about 340, of whom nearly one-third are criminals. Tobacco is freely allowed in this asylum, and the wards are consequently strongly tainted with its smoke. The servants appear to participate in the privilege. This differs widely from the discipline of the American asylums.

I visited the Hanwell Asylum, near London, twice; on both occasions examining minutely the condition of the patients, and the arrangements and discipline of the institution. The chief medical officer, Dr. Begley, has been 22 years in this Asylum, and the appearance of everything about it indicates that his duties are well performed.

Hanwell is the second largest asylum in England, and now contains about 1200 patients. The grounds are extensive, and are much ornamented by shrubberies and flowers. The buildings are large, and complete in their arrangements, affording abundant means of classification. Dr. Begley very kindly caused the clerk of works to draw for me a ground plan of the buildings, which I submit to your inspection. In this asylum, as in all those of Europe, in the vicinity of large towns, the number of cases of that peculiar form of insanity, designated "general paralysis," characterized by impairment of muscular power, and ultimately by its total extinction, and by mental delusions of an exalted and very distinct order, is very considerable. It is there, as on this continent, almost exclusively confined to males; but in America it is comparatively rare in either sex. I have never seen a case in a female in the American asylums. In the large asylums of London, Wakefield, Lancaster and Dublin, in which its victims form seperate groups, in distinct apartments, their inspection is, to the professional visitor, a painful task. He knows that they are beyond the reach of remedial agents. Not unfrequently it lays hold of men of distinguished energy, and eminent position. In Scotland, the asylum physicians seem to regard it as largely ascribable to intemperance. The experience of this country leans rather to an opposite conclusion, as the majority of its victims here have been men of temperate habits. Its almost restricted incidence to the male sex, might suggest some relation between the malady and sexual propensity; but it might be both unjust and dangerous to venture further than conjecture. The general comfort and tranquility of the patients of Hanwell are very pleasing to visitors. It has constantly been remarked by American superintendents, in their tours of inspection, that the inmates of European asylums are much more tractable, quiet and orderly, than those of American asylums. This statement is quite correct as to the English asylums, but not as to those of Ireland or Scotland. In the latter two countries I found the patients as noisy, restless, and mischievous, as those of American asylums. The people of England, as well as the inmates of their lunatic asylums, use a generous diet, and free beverages of ale; and there is some constitutional affinity between good feeding and mental composure. Be the fact as it may, there is more scolding in the Scotch, and more restlessness and mischief in the Irish asylums, tenfold, than in those of England.

The largest lunatic asylum in England, is that of Colney Hatch, six miles north of London. It now lodges about 2000 patients, of whom 800 are men, and 1200 women. The grounds contain about 140 acres, and the main building has a front extension one-third of a mile, with numerous wings projecting from the rear.

The height, including basement, which is not excavated, is three stories. The establishment is complete in every requisite, and has cost £500,000 sterling. My attention was given chiefly to the female division, as the medical officer of the male side was absent. The females are divided into 21 classes. The result of this extended classification is, that good order and general comfort prevail; and the task of supervision is by no means so difficult as those who have never visited such an institution might suppose. A few distinct divisions in any lunatic asylum, are found adequate to relieve the great majority from annoyance and disquietude; whilst in an asylum with ever so small a number of inmates, where the noisy, violent, obscene, filthy and idiotic are, from lack of distinct accommodation, mixed with other classes, there can be neither peace, comfort, nor safety. In this asylum, as in every other in England, great importance is attached to the ornamenting of the grounds, and the interior of the house; and every possible means of employing and amusing the patients is had recourse to. I do not think it is advisable to found lunatic asylums on a large scale; but the authorities of this institution have avoided the far more serious error of leaving it incomplete. Had they erected no more than its large front wards, it would now be in a sad condition, and would be pointed to as a proof of the impropriety of large foundations for the insane.

Having satisfied myself with the inspection of the leading insane institutions of the metropolis, I set out for a further examination of the provincial ones; and on the 7th of July, visited that of Shrewsbury, Shropshire. This asylum has only 30 acres of land; but the site is very beautiful, and the soil is good. The farm is well tilled by spade labor. There were 349 patients in the institution when I visited it. An additional detached building has recently been opened for patients, but the original building was complete prior to this erection. The asylum is an excellent institution; but nothing connected with it is more attractive than its medical chief, Dr. Oliver, whose whole deportment and conversation, both among his patients and in the domestic and social circles, evinced goodness of heart and clearness of intellect.

Dr. Oliver has, for some years, pursued a heroic line of treatment in certain forms of acute insanity, in which his medical confreres tremble to follow him. I refer to his profuse exhibition of opium, which he informed me he administers not only with impunity, but with signal benefit, to the extent of 20 or 25 grains twice in the 24 hours. It certainly would be unadvisable for an asylum physician in this country, where the medical profession is not altogether composed of gentlemen, nor of extensive readers, to venture on such bold practice; more especially, too, as coroners' inquests are now objects of keen competition.

The next asylum visited by me was that of Chester, which is an old foundation, and consequently a defective one. Important improvements and extensions are now in progress, which will raise the capacity of the institution from 200 to 500 patients. In this asylum were, until recently, to be found almost all the structural faults of the former age; as strong, narrow cells, ponderous doors, iron bars and gratings, high, prison walls, stone floors, and numerous other precautionary provisions against real or imaginary dangers. The work of removal and transformation has gone on slowly, and by piece-meal, like other salutary reforms; but even now a few vestiges of the olden times remain to demonstrate the value of magisterial conservatism. The medical superintendent has not totally succeeded in introducing as orthodox truths in Chester, facts which have been ratified by the experience of nearly all the world outside.

Until lately this asylum had only eleven acres of land; an addition of 44 acres has now given it a very good farm. Faulty buildings test the capacity of a superintendent; and Dr. Brushfield has shown how much can be accomplished, under even the greatest disadvantages; yet his hardest work has not been in the management of his patients, but in the slow conversion of his superiors. But however reluctant these have been in improving the asylum, they have shown great consideration for their superintendent; for they have erected for him a very handsome and capacious residence, separate from the asylum.

Having inspected the Chester Asylum, I proceeded, on 8th July, by Holyhead to Dublin; and as I had been informed in England that I would find a very good asylum at Killarney, I went at once to this famous place. I found the institution superior to my expectations. Certainly had I not seen it, I could never have believed that contiguous to such a den of filth, laziness, and unaspiring poverty, as the old town of Killarney presented to my organs of sight and smell, so pretty, clean, and comfortable an asylum could be established or continued. The building has been erected under the instructions of the Irish Board of Works, and it does infinite credit to the judgment and good taste of this body. It is fortunate for the poor of Ireland, that a central authority like this exists, to control erections of public utility; forfrom all I could gather of the views and wishes of the resident gentry and proprietors, it would be many centuries yet before their conceptions of the wants of the insane poor would carry them to the establishment of so good an institution as the Killarney Asylum. It has cost only £40,000 sterling, and has 222 beds, of which about four-fifths are occupied. The annual expense for 1856, was under £3,500 sterling, yet the gentry complain of the institution as extravagant. It will be an arduous and very thankless task to keep this institution up within a decent distance of the present status of asylums elsewhere.

The Killarney Asylum is doing more good than in the mere care and cure of the insane inmates. It is a model school of neatness and good order for the instruction of the people. I inquired of Dr. Lawlor, the superintendent, whence he obtained his servants. I could not think they could be drawn from the contiguous population; but he told me they were, and that he had trained them all. I thought his office must be no sinecure. He also told me that his patients, when recovered, all went out greatly improved in habits, and proved more useful than they ever had been before their insanity. Dr. Lawlor is doing a great and useful work, in this demonstration of the capability for improvement of a most unpromising class of people, and the Irish government deserves high commendation for the establishment of this institution, and various others of similar merit. The sleeping rooms of the Killarney Asylum are placed on one side only of the corridor, so that abundant light and free ventilation are commanded.

The site is one of the best which this picturesque country presents, and every part of the building has been constructed with scrupulous regard to neatness, comfort, and convenience. I found infinitely more pleasure in the inspection of this institution than in the boasted beauty of the adjacent lakes and mountains of Killarney.

On Monday, 10th July, I returned to Dublin, to inspect the celebrated Richmond Asylum. This institution, like that of Wakefield, consists of two distinct buildings, an old and a new one. I wish I could say that in other respects it resembled its English sister. The grounds, being within the city, are very limited, and the buildings are both over-crowded. The total number of patients is 650. The medical superintendent appears to occupy a very indefinite position. He has no resident assistant; but several salaried officers, designated visiting physicians, attend daily, and record their visits. Of course they also append their signatures to the quarterly pay-lists;—and here, perhaps, it would be as well their function ceased; for neither in this asylum, nor any other in which similar appointments exist, could I discover any advantage in the regulation, but very much to the contrary. The treatment of the insane must be conducted, and can be efficiently conducted, only by medical officers constantly residing amongst them; and every interference by other parties, whether with the patients or with the servants, must prove pernicious. In the best managed asylums of the old country, where these visiting physicians still continue to be appointed, their duties are, practically, a nullity; in those in which they exceed this, the function of the superintendent approximates to nullity, and the institutions suffer accordingly. The Richmond Asylum will most probably remain as it is for a long time to come. It is too near the Liffey, and too far from the Thames.

On the 13th July, I left Dublin for Belfast; and here I found an insane asylum which may compare advantageously, except in its diet-tables, with the best in England. Dr. Stewart is the very life and spirit of his institution. He seems to live for nothing else; and everything in and around his establishment bears the impress of his energy and good taste. His services are duly appreciated by the Board of Governors, and the intelligent community of Antrim and Down. The number of patients now in the asylum is 360. The want of increased accommodation is much felt, and great hardship is suffered by the excluded insane and their families.

The next asylum visited by me was that of Armagh. This, I

trust, is not only the worst in Ireland, but in all the world. It contains only about 150 patients, yet is the sole insane asylum for the three populous counties of Armagh, Tyrone, and Monaghan. The arm of paternal despotism is wanted here; and it is to be hoped that the Irish Board of Works will, ere long, do for these counties what it has done for Kerry. Nothing short of arbitrary central power will be adequate. The landed proprietary, who compose the grand juries, set their faces against local imposts. The claims of humanity are but as dust in the balance, against the cravings of landlords. The Armagh Asylum is crammed. There is not a water-closet in the building, and doorless privies in the walls of its prison-airing courts require no sign-board to indicate their location. When it is requisite to clean out these receptacles, the offensive matter has actually to be carried through the asylum. Water, it may be said, there is none, though the city main passes close to the premises. The foul air of the rotten, dungeon-basement, is felt throughout the house.—The quantity of land is eight acres. This is in Christian Ulster.

Having spent a short time among the few remaining friends and companions of my boyhood, I left my native land for Scotland, where I wished to inspect the asylums of Glasgow, Edinburgh, and Dumfries. I found much to admire in each of these institutions, and observed a few things requiring improvement. Each, like those of Wakefield and Dublin, consists of two distinct buildings, an old and a new. The old buildings are instructive, as showing the faults and defects of the past, and the new, as exhibiting, in contrast, the improvements of the present time.

The site of the Glasgow Asylum, at Gartnavel, is truly beautiful, and the arrangements and discipline of the institution are generally excellent. Here the new building is appropriated to the higher order of patients, many of whom pay high rates of board. In Edinburgh and Dumfries, the new buildings have been appropriated to the poor patients. The insane in the Scotch asylums are treated in the same gentle and kind manner as those in England; but there is a very marked difference in their demeanor; they are clamorous and discontented, and some of them abuse the superintendent and his assistants severely and loudly. Their scolding is all borne with exemplary patience, and probably they are benefited by these occasional bursts of pent-up eloquence.

The total number of patients in the Glasgow Asylum is 520. The means of classification are ample, and consequently the general comfort of the inmates is very satisfactory. Dr. McIntosh speaks to his patients in the most mild and conciliatory manner, and appears to study very carefully their peculiar mental tendencies and caprices.

In the Edinburgh and Dumfries asylums I found little different from what I observed at Glasgow. In all three the number of cases of general paralysis was painfully large. The medical officers seemed disposed to charge the evil to intemperance.

The profuse use of tobacco and snuff, in Scotland, might perhaps justly come in for a share of the accusation. I was surprised to observe the extent to which this costly drug is consumed in that country of common use. Dr. McIntosh informed me that in his institution hereditary insanity is strikingly common. The grounds of the Glasgow asylum are 70 acres; those of Edinburgh, 67; and those of Dumfries probably about as much.

The new asylums at Edinburgh and Dumfries stand a short distance from the old ones, from which they are screened by handsome intervening shrubberies. Besides the usual number of wards in the Edinburgh new building, two appended buildings, of one story, are placed a short distance from the ends, for the lodgment of noisy and other troublesome patients. These apartments are of great value to the institution. Ventilation is imperfect in the Edinburgh and Dumfries asylums.

The last asylum visited by me was that of Lancaster, which is one of the three establishments supported by the county of Lancashire, containing an aggregate of over 1600 patients, of which the Lancaster asylum has 724. I was prepared, by previous information, to find this institution one of high merit; yet it surpassed my expectations. The quanity of land is only fifty-seven acres. The grounds are laid out with true English taste, and are kept scrupulously neat. The building was orginally of the H form, but by various additions has now lost its early aspect; yet all the arrangements are judicious. The laundry, and drying and ironing rooms are very extensive. The washing is done by hand, and gives employment to a large number of female patients. The kitchens are large, and very complete in apparatus. Every part of this establishment, and everything within and without, in as clean as a new pin. It is a perfect model of English neatness, English comfort, and English industry.

A large, two-story, stone building, detached from the chief building, has been recently erected at a cost of only £1500, for the residence of 50 male patients, of various trades. The workshops are in the first story, and the eating and sleeping-rooms in the second. When in the shoemaker's shop, I enquired whether any casuality had ever occurred there, from the presence of dangerous implements, and was informed that one patient had injured another by a blow with a shoe-hammer; it was then pointed out to me that all the hammers were now chained to the seats, "by order of the Governors." I enquired, "What of the knives?" but was answered that no patient had yet hurt any one with a knife. Thus we provide against an evil which has occurred; yet, are fearless of much greater, because it has not yet happened.

Dr. Broadhurst stated that among the patients of this asylum general paralysis is very common; and that the same fact obtains in the other asylums of Lancashire. He informed me that, in the few instances in which the disease had presented itself in females, it was not accompanied by the peculiar mental delusions of ambitious monomania, which almost invariably manifest themselves in male cases. I should regard this fact as a proof of the non-identity of the male and female maladies. The floors and doors of this asylum are all of British and American oak, and are as perfect as when made. The stairs are of stone, and are already so much worn as to require replacement.

Whilst waiting for the departure of the steamer at Liverpool, I availed myself of the kindness of Dr. Archer, surgeon to the Liverpool Burrough prison, to inspect this institution. The establishment had, when I visited it, about 1000 prisoners, the majority of whom were convicts. It has sometimes had nearly 1,200 prisoners. It is about double the size of the Wakefield new prison; but its arrangements and discipline are on the same principles. The ventilation is on the same plan as in Wakefield; but in the summer the tower-fires are not kept going as in Wakefield. The difference of the air in the two buildings was to me very perceptible, and proved that the system is efficient, but must not be intermitted.

Insanity occurs in this prison annually to the extent of about 20 cases. Dr. Archer appears, from his reports, to regard the malady as frequently arising from solitary confinement. In such cases, association has been found a successful remedy.—*American Journal of Insanity.*

